# Inter- and Intrapersonal Associations Between Physiology and Mental Health: A Longitudinal Study Using Wearables and Mental Health Surveys

**DOI:** 10.2196/64955

**Published:** 2025-07-23

**Authors:** David Presby, Summer Jasinski, Emily Capodilupo, Kristen E Holmes, William von Hippel, Gregory J Grosicki, Victoria Lee

**Affiliations:** 1 Department of Computational Biology, University of Lausanne, Genopod, Lausanne, 1000, Switzerland, 41 216921111; 2 Swiss Institute of Bioinformatics, Lausanne, Switzerland; 3 Department of Research, Algorithms, and Data Science, WHOOP Inc, Boston, MA, United States; 4 Department of Performance Science, WHOOP Inc, Boston, MA, United States; 5 School of Psychology, University of Queensland, Queensland, Australia; 6 Research with Impact, Brisbane, Australia

**Keywords:** digital health, wearables, intrapersonal, interpersonal, physiology, mental health, longitudinal study, awareness, digital health technologies, web-based applications, mHealth, mobile health, apps, applications, smartphones, mobile phone

## Abstract

**Background:**

More than 1 in 8 people potentially live with a mental health disorder, yet fewer than half receive treatment. Poor mental health awareness may contribute to this treatment gap, and digital health technologies, like wearables and their associated phone- and web-based applications, have the potential to reduce the mental health awareness gap due to their ease of adoption, objective feedback, and high rate of engagement.

**Objective:**

This study aimed to better understand the relationships between mental health and objective wearable-derived metrics.

**Methods:**

We examined the longitudinal results of monthly mental health surveys (Patient Health Questionnaire-2, Generalized Anxiety Disorder 2-item, and Perceived Stress Scale) delivered over 13 months to 181,574 individuals wearing a device (WHOOP, Inc.) that measures sleep, cardiorespiratory parameters, and physical activity (up to 307,860 survey responses and 7,942,176 days of total wear time). Generalized linear mixed models, cross-lag analyses, and intrapersonal scaling were used to assess interpersonal and intrapersonal associations between wearable-derived metrics and mental health outcomes. Age, gender, BMI, and time of year were used as covariates in the models.

**Results:**

Interpersonal associations between wearable-derived metrics and mental health outcomes indicate that individuals with better sleep characteristics (ie, longer sleep durations and more consistent wake and sleep times), higher heart rate variabilities (HRV), lower resting heart rates (RHR), and higher levels of physical activity report lower levels of depression, anxiety, and stress. Intrapersonal associations between wearable-derived metrics and mental health outcomes displayed similar results as the between-person analyses, with higher HRVs, lower RHRs, and more physical activity generally coinciding with improved mental health outcomes. However, intrapersonal wearable-derived sleep metric associations diverged from the interpersonal association findings when specifically looking at sleep duration and depression, whereby increased sleep durations within an individual were associated with higher levels of depression. In interpersonal analyses, the largest association observed was between the Perceived Stress Scale scores and RHR, with a standardized coefficient of 0.09 (*P*<.001); in intrapersonal analyses, the largest association observed was between the Patient Health Questionnaire-2 scores and summated heart rate zones—a proxy for physical activity—with a standardized coefficient of −0.04 (*P*<.001). Cross-lagged models demonstrated that higher levels of reported stress preceded higher RHRs, respiratory rates, and sleep duration variabilities, as well as lower HRVs.

**Conclusions:**

Overall, this investigation reveals that numerous physiological variables measured by wearables are associated with mental state in free-living environments. These findings underscore the potential of wearable-derived physiological and behavioral monitoring to serve as objective complements to traditional subjective assessments in mental health research and care. However, given the complex nature of mental health disorders, further research is needed to determine how these metrics can be effectively integrated into clinical practice.

## Introduction

In 2019, the World Health Organization estimated that 1 in 8 people were living with a mental health disorder [1], with the prevalence of anxiety and depression—the most common mental health disorders—estimated to have increased by 26% and 28%, respectively, during the COVID-19 pandemic [2]. Compounding these issues, fewer than half of individuals living with mental illness receive treatment [3]. The societal impact of untreated mental health issues extends beyond individual well-being to encompass substantial economic burdens as well [4], reinforcing the importance of treatment for mental health disorders. One reason for widespread undertreatment is poor mental health literacy [5], raising the possibility that broadened access to mental health diagnostic tools may help to reduce the burden of mental health disorders.

A promising avenue for expanding awareness into individual mental health status can be found in digital technologies like smartphone apps and wearables. Although digital technology adoption may be limited by digital literacy and socioeconomic status [6,7], around half of all smartphone owners use a health and wellness app [8,9] and, in a survey dispersed to a population reflective of the US population, 44.5% (10,679/23,974) of respondents indicated they owned a wearable device [10]. In addition, wearables may track physiological measures, like sleep and cardiovascular parameters, that have been shown to associate with mental health outcomes [11-15], suggesting wearables have the potential to provide real-time feedback on physiological states predictive of mental health status. Despite the prevalence of research investigating the relationships between mental health and physiological measures, little is known about how wearable-derived measures may reflect mental health status in free-living environments.

In this investigation, we provided members of a commercial wearable device (WHOOP, Inc.) with a monthly survey that gauged depression, anxiety, and stress via the Patient Health Questionnaire-2 (PHQ2), Generalized Anxiety Disorder 2-item (GAD2), and Perceived Stress Scale (PSS), respectively. We combined these mental health data with physiological and behavioral data from the wearable device – including measures of sleep, cardiorespiratory function, and physical activity – to better understand the relationships between mental health status and aspects of behavior and physiology. Through this interdisciplinary approach, we aimed to elucidate nuanced connections between mental health status and wearable-derived sleep, cardiorespiratory, and physical activity parameters to assess the potential utility of wearable technology in advancing our understanding and management of mental health issues.

## Methods

### Data Collection

Responses to mental health questionnaires were collected via monthly surveys delivered through the WHOOP mobile app to all interested WHOOP members over a 13-month period, from March 2022 to April 2023. The mental health assessment questions included the PHQ2 [16,17] (a questionnaire meant to gauge depression), the GAD2 [18] (a questionnaire meant to gauge anxiety), and the PSS [19] (a questionnaire meant to gauge perceived stress). The questions and their responses are available as Multimedia Appendix 1. To account for the different response scales between the PHQ2 or GAD2 and PSS, the PSS was linearly scaled from 0 to 6. The combined responses from the PHQ2, GAD2, and PSS assessments were then summated as a measure of overall mental state, and this score is referred to as the combination score. The scores of the PHQ2, GAD2, and PSS reflect severity or likelihood of a mental health disorder (eg, scoring higher on PHQ2 means a greater likelihood of being depressed or an increased severity of depression) [16,17,20].

A wrist-worn device (WHOOP strap version 3.0 and 4.0) that continuously collects heart rate (via photoplethysmography) and accelerometry (via 3-axis accelerometer) data was used to calculate sleep, cardiorespiratory, and physical activity. Sleep measures included total sleep duration, sleep efficiency, SD of sleep duration, sleep consistency, SD of wake times, and SD of sleep times. Sleep consistency is adapted from the sleep irregularity index and is calculated as the percentage of concordance when individuals are in the same state (sleep vs awake) at different time points over a 4-day interval, with comparisons of intervals further apart being assigned progressively lower weights [21,22]. A higher sleep consistency value reflects a lower variability in sleep-wake timing. Cardiorespiratory measures included resting heart rate (RHR), average heart rate variability (HRV_av_), coefficient of variation of heart rate variability (HRV_cv_), and respiratory rate. RHR is calculated as a weighted mean of heart rate during sleep. Heart rate variability (HRV) is calculated as the weighted average of the root-mean-square of successive differences of the interbeat intervals during sleep. For RHR and HRV, higher weights were assigned to periods with a higher probability of slow wave sleep and periods closer to the end of the sleep. Respiratory rate is calculated as the median of respirations per minute calculated via the interbeat intervals throughout sleep. Heart rate zones (HRZs) were determined as a percentage of the participant’s estimated maximal heart rate, where zone 0<50% of maximal heart rate, zone 1=50%‐60%, zone 2=60%‐70%, zone 3=70%‐80%, zone 4=80%‐90%, and zone 5=90%‐100%. Physical activity measures included percent time spent in HRZ 1‐5, summated HRZs, and physical activity level (total energy expenditure divided by resting energy expenditure). Summated HRZ was calculated as the time spent in each HRZ, in minutes, multiplied by the corresponding factor for each zone and then summated. Physical activity level was calculated as the total calories expended divided by resting energy expenditure. Cardiovascular, respiratory, and sleep measures derived throughout sleep have been validated against gold standard polysomnography measures and have been found to have a low degree of bias and low precision errors (eg, <20 min bias and precision errors for sleep duration; 0.7 beats per min bias for heart rate; 4.7 ms bias for HRV; and 1.8% bias for respiratory rate) [23-25]. More details on the methods for calculating wearable-derived metrics can be found in Multimedia Appendix 2.

### Data Filtering and Aggregation

Survey responses from individuals older than 21 years of age were considered for this study. Each survey response from the participant over the 13-month period was paired with the wearable data from the 14 or 28 days preceding the survey response. Furthermore, 14 days of wearable data leading up to the response were used for the PHQ2 and GAD2 as the questions required participants to reflect on how they felt over the previous 2 weeks; 28 days of wearable data leading up to the response were used for the PSS as the questions required participants to reflect on how they felt over the previous month. Except for the measures that pertained to variability, the daily measures from the time leading up to completing a survey were averaged via the mean. For measures pertaining to variability, except for sleep consistency, the sample SD of the daily measures over the relevant period of time was used. Periods during which a user had recorded fewer than 7 days of wearable data in either the 14- or 28-day period leading up to completing their survey were excluded from analysis. Participants were included if they selected man or woman when asked to self-identify their gender. Extreme values were clipped at 17 and 40 kg/m^2^ for BMI and at 70 years for age. BMI was also examined by binning into classification ranges: underweight (<18.5 kg/m^2^), healthy weight (≥18.5 kg/m^2^ and <25 kg/m^2^), overweight (≥25 kg/m^2^ and <30 kg/m^2^), and obese (≥30 kg/m^2^). As this investigation included participants from both the northern and southern hemispheres, which experience opposite seasons for a given date, we adjusted the submit month of participants from the southern hemisphere by shifting each submit month to 6 months forward. For example, responses submitted in June from participants in the southern hemisphere were adjusted to December to align the seasons of the northern and southern hemispheres.

A subsample of individuals was used to understand the intrapersonal relationships and the relationship between physical activity, estimated via HRZs, and mental health status. Eligibility for this subsample required that participants responded to the survey at least 8 times and recorded more than six 24-hour periods containing at least 1000 minutes of data in the time frame leading up to each of the survey responses.

### Association Analysis

Generalized mixed linear models were used to examine the relationships between mental health and age, gender, BMI, and month of submission. In these models, the mental health outcome was modeled as the dependent variable; age, gender, BMI, and month of submission were treated as the independent variables; and each participant was treated as a random effect. Natural cubic splines were used for BMI and month of submission due to the nonlinear relationships these variables displayed with mental health. Estimated marginal means for age, gender, BMI, and month of submission were extracted from the models to provide estimates and variances for plotting. To examine the effects of different BMI categories and seasons on mental health, pairwise comparisons were made using the emmeans package in R (R Core Team).

Generalized mixed linear models were also used to examine the relationships between mental health responses and wearable-derived metrics. In these models, mental health outcomes were modeled as dependent variables, and the wearable-derived metrics as the independent variable. Age, gender, BMI, and the month of submission were used as covariates in the model, and each participant was treated as a random effect. For intrapersonal associations, wearable-derived metrics were person-mean-centered. Natural cubic splines were used for BMI and month of submission due to the nonlinear relationships these variables displayed with mental health and wearable-derived metrics. The raw and standardized coefficients (β) from the models were extracted to describe the relationship between wearable metrics and mental health.

### Cross-Lag Analysis

To examine the temporal relationships between mental health outcomes and physiological variables, we conducted a lagged analysis using structural equation modeling via the lavaan package in R. For these models, both mental health outcomes and physiological variables were centered within each participant by subtracting their respective within-person means. Lagged variables were generated for both mental health outcomes and physiological variables by using the immediate preceding mean person-centered observation. We accounted for the nested structure due to repeated measures within participants by using the built-in clustering functionality. To flexibly model time of year effects, natural spline transformations with 3 degrees of freedom were applied to the submission month. Demographic variables—such as age, BMI, and gender—were assumed to be invariant over time and modeled as such. BMI and age were further transformed using natural splines (with 3 degrees of freedom) to capture potential nonlinear effects in the relationships with the outcomes. Autoregressive effects were modeled by regressing each outcome on its own lagged value. Contemporaneous covariance was addressed by allowing for residual correlations between the mental health variable and physiological variable at the same time point. Reciprocal influences were examined by regressing the mental health variable on the lagged physiological variable and vice versa, enabling an exploration of bidirectional effects. Cross-lagged paths were estimated within the structural equation model framework, with standardized parameter estimates extracted to facilitate interpretation of temporal relationships (simplified diagram of cross-lag design in Multimedia Appendix 3).

The significance threshold for all analyses was set to 0.001.

### Ethical Considerations

Participants consented to their anonymized data being used for research purposes. Since data were not identifiable and were stored on a secure server, this study was deemed exempt from institutional review board oversight by Advarra’s Institutional Review Board (Columbia, Maryland). Participants did not receive compensation for their participation in this study.

## Results

### Description of the Study and Sample

**Figure 1.**
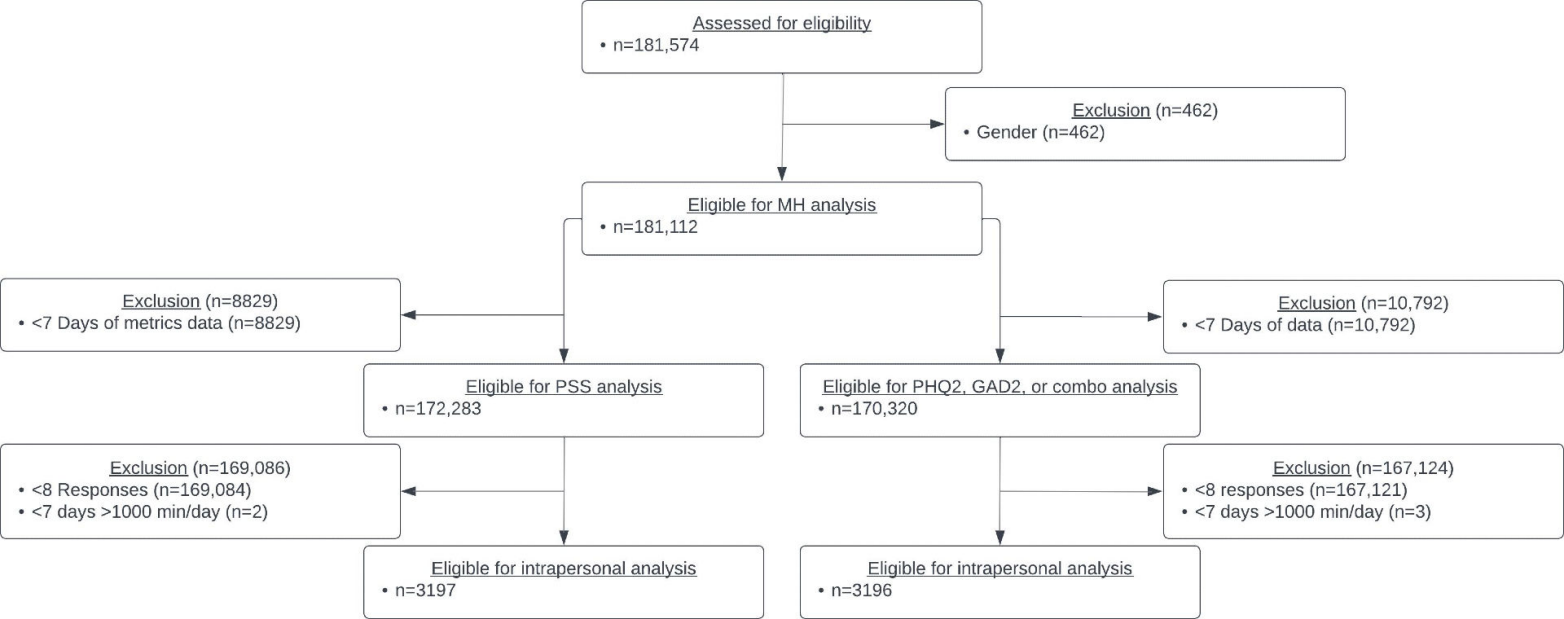
Flowchart of study design. After initial inclusion criteria based on gender and age, inclusion was individually assessed for the Patient Health Questionnaire-2, Generalized Anxiety Disorder 2-item, or combination score analyses and the Perceived Stress Scale analyses, which required at least 7 days of metrics. Mental health assessments were independently filtered due to the number of days included in each assessment, whereby the Patient Health Questionnaire-2, Generalized Anxiety Disorder 2-item, combination score analyses were based on 14 days' worth of metrics leading in, while the Perceived Stress Scale analyses were based on 28 days' worth of metrics leading in. For the intrapersonal and physical activity analyses, data were further filtered to remove participants that had responded to the survey less than 8 times and to remove participants that had less than 7 days of metrics where a participant wore the strap for more than 1000 minutes over a 24-hour period. combo: combination score; GAD2: Generalized Anxiety Disorder 2-item; MH: mental health; PHQ2: Patient Health Questionnaire-2; PSS: Perceived Stress Scale.

A total of 181,574 individuals were considered for eligibility, and, after excluding due to either gender and data availability, 172,283 individuals were deemed eligible for the PSS analysis and 170,320 were considered eligible for the PHQ2, GAD2, and combined score analyses. Among the subset of individuals included in mental health and physical activity association analyses, 3197 participants were included for the PSS, and 3196 were included participants for the PHQ2, GAD2, and combination score analysis (Figure 1). The number of days included in each analysis and demographic information for each subgroup are provided in Table 1.

**Table 1.** Characteristics of study participants.

Characteristic	GAD2^a^, PHQ2^b^, or combo^c^		PSS^d^	
	Interpersonal (N=170,320^e^)	Intrapersonal (N=3196^e^)	Interpersonal (N=172,283^e^)	Intrapersonal (N=3197^e^)
Total days of metrics, n	4,123,851	468,841	7,942,176	933,161
Average days of data leading up to survey, mean (SD)	13.2 (1.5)	13.8 (0.3)	24.7 (5.1)	27.4 (0.8)
Number of times responded to survey, mean (SD)	1.84 (1.79)	10.68 (1.52)	1.83 (1.78)	10.68 (1.52)
Gender, n (%)				
Women	56,745 (33)	1174 (37)	57,455 (33)	1174 (37)
Men	113,575 (67)	2022 (63)	114,828 (67)	2023 (63)
Age, mean (SD)	37.39 (10.36)	40.71 (10.80)	37.36 (10.36)	40.71 (10.80)
BMI, mean (SD)	26.35 (10.42)	25.61 (4.08)	26.40 (10.38)	25.72 (4.15)

a GAD2: Generalized Anxiety Disorder 2-item.

b PHQ2: Patient Health Questionnaire-2.

c combo: combination score.

d PSS: Perceived Stress Scale.

e Sum.

### Associations Between Mental Health and Age, Gender, BMI, and Time of Year

Self-reported mental health scores decreased with age (indicating better mental health) across all mental health outcomes (Figure (a) in Multimedia Appendix 4; PHQ2: *P*<.001, standardized β coefficient=−0.019; GAD2: *P*<.001, standardized β coefficient=−0.023; PSS: *P*<.001, standardized β coefficient=−0.019; combined: *P*<.001, standardized β coefficient=−0.06). On average, males reported better mental health on their assessments than females (Figure (a) in Multimedia Appendix 4). Mental health scores shared a nonlinear relationship with BMI, whereby mental health was worse at the upper (ie, obese) and lower (ie, underweight) extremes as compared with a healthy or overweight BMI (Figure (b) in Multimedia Appendix 4; all *P*<.001). Mental health scores also displayed a seasonal pattern, whereby individuals reported worse depression in the Fall as compared with all other seasons and worse stress in the Fall as compared with Spring and Winter (Figure (c) in Multimedia Appendix 4; all *P*<.001). Pairwise comparisons among BMI groups and seasons can be found in Multimedia Appendix 5. Due to the associations observed between demographics and seasonality with mental health outcomes, we incorporate demographics and seasonality as covariates in our models examining the relationships between physiological variables and mental health outcomes.

### Wearable Derived Metrics Associate With Mental Health Assessments

#### Sleep Metrics

**Figure 2.**
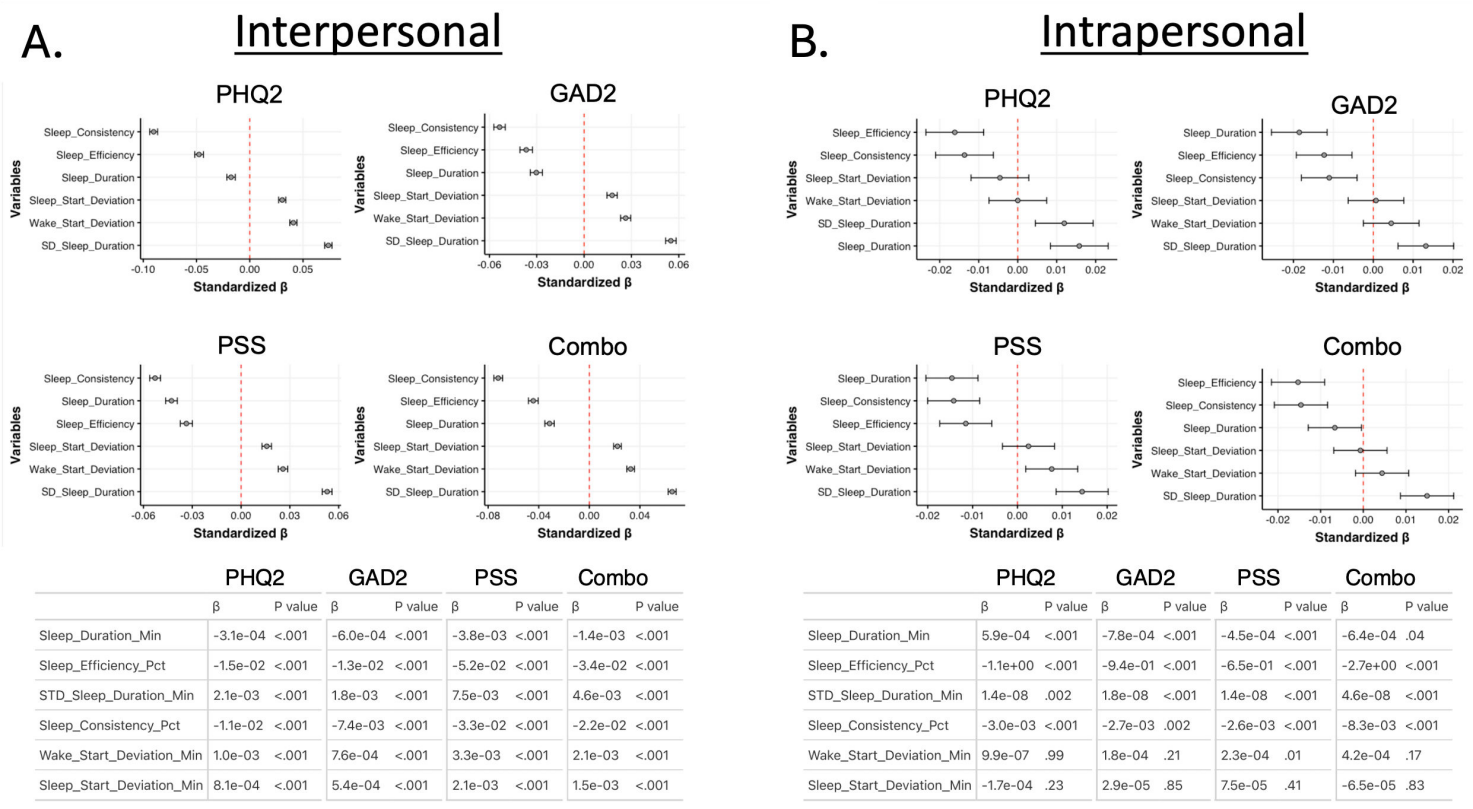
Inter- and intrapersonal associations between sleep and mental health. (A) Interpersonal and (B) intrapersonal associations between sleep and mental health. For both (A) and (B), the top left graph reflects the associations with depression (Patient Health Questionnaire-2), the top right graph reflects associations with anxiety (Generalized Anxiety Disorder 2-item), the bottom left graph reflects associations with stress (Perceived Stress Scale), and the bottom right graph reflects associations with overall mental health, assessed by taking the summation of the Patient Health Questionnaire-2, Generalized Anxiety Disorder 2-item, and Perceived Stress Scale assessments (combination score). Graphs are generated using standardized coefficients, and error bars reflect 95% CIs. The tables below the graphs in (A) and (B) display the raw coefficients (β) along with the parameters’ associated *P* value for each sleep metric association with each mental health outcome. combo: combination score; GAD2: Generalized Anxiety Disorder 2-item; Min: minute; Pct: percentage; PHQ2: Patient Health Questionnaire-2; PSS: Perceived Stress Scale.

When compared between persons, higher amounts of sleep duration, sleep efficiency, and sleep consistency were associated with better mental health, whereas higher levels of variance in sleep duration, time of waking, and time of falling asleep were associated with worse mental health, as assessed by reported scores on the depression, anxiety, stress, and combined scales (Figure 2A). When compared within persons, reductions in sleep efficiency and sleep consistency were associated with worse mental health outcomes (Figure 2B). Notably, increased sleep duration within individuals was associated with worse scores on the depression assessment and better scores on the anxiety and stress assessment (Figure 2B).

#### Cardiorespiratory Metrics

**Figure 3.**
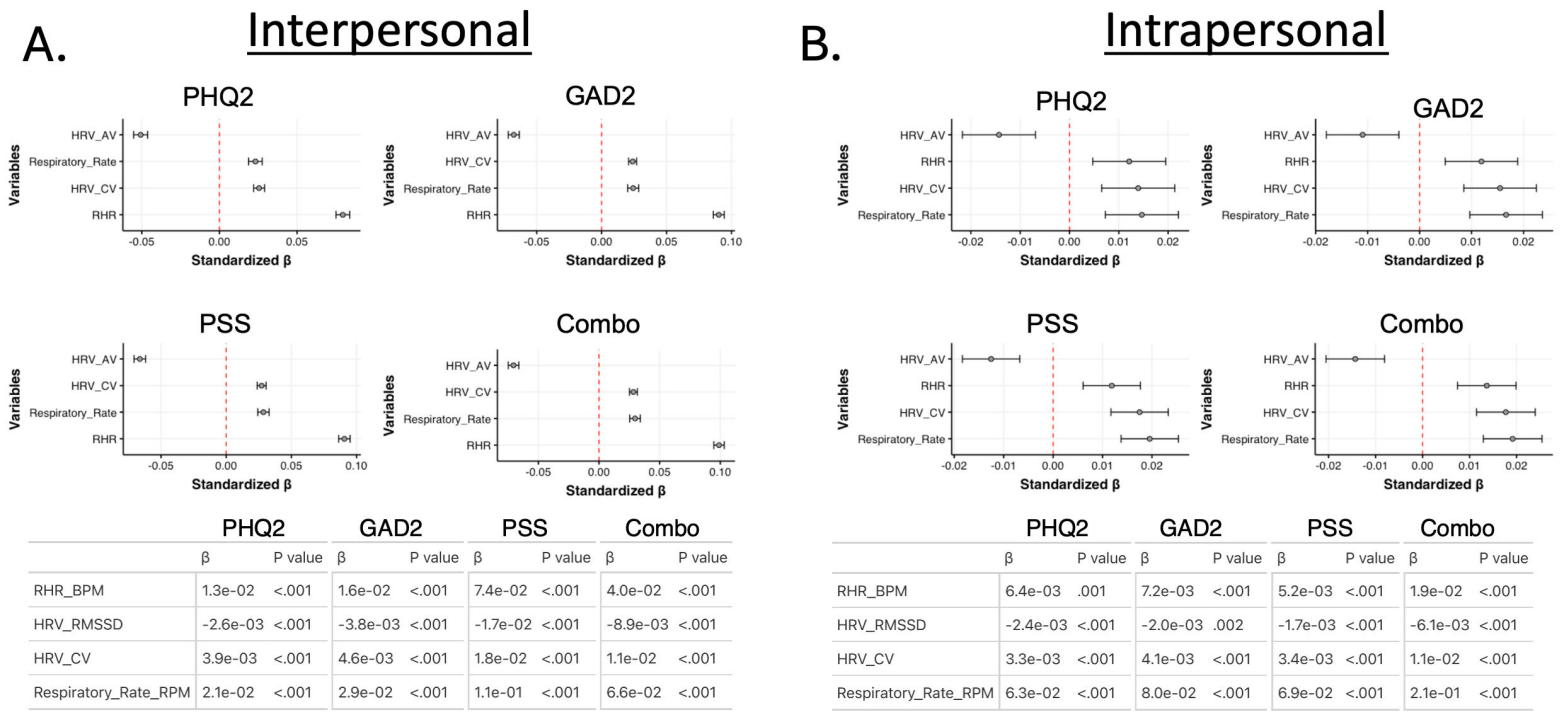
Inter- and intrapersonal associations between cardiorespiratory metrics and mental health. (A) Interpersonal and (B) intrapersonal associations between sleep and mental health. For both (A) and (B), the top left graph reflects the associations with depression (Patient Health Questionnaire-2), the top right graph reflects associations with anxiety (Generalized Anxiety Disorder 2-item), the bottom left graph reflects associations with stress (Perceived Stress Scale), and the bottom right graph reflects associations with overall mental health, assessed by taking the summation of the Patient Health Questionnaire-2, Generalized Anxiety Disorder 2-item, and Perceived Stress Scale assessments (combination score). Graphs are generated using standardized coefficients, and error bars reflect 95% CIs. The tables below the graphs in (A) and (B) display the raw coefficients (β) along with the parameters’ associated *P* value for each sleep metric association with each mental health outcome. AV: average; BPM: beats per minute; combo: combination score; CV: coefficient of variation; GAD2: Generalized Anxiety Disorder 2-item; HRV: heart rate variability; PHQ2: Patient Health Questionnaire-2; PSS: Perceived Stress Scale; RHR: resting heart rate; RMSSD: root-mean-square of successive differences; RPM =Respirations per minute.

For both inter- and intrapersonal associations, higher HRV_av_ values were associated with better mental health outcomes (Figure 3A and 3B). Furthermore, for both inter- and intraperson associations, lower values of RHR, HRV_cv_, and respiratory rate were associated with better mental health (Figure 3A and 3B).

#### HRZs

**Figure 4.**
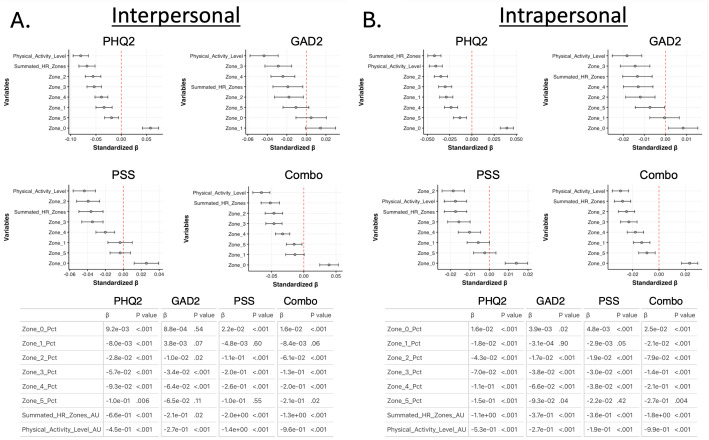
Inter- and intrapersonal associations between physical activity measures and mental health. (A) interpersonal and (B) intrapersonal associations between physical activity and mental health. For both (A) and (B), the top left graph reflects the associations with depression (Patient Health Questionnaire-2), the top right graph reflects associations with anxiety (Generalized Anxiety Disorder 2-item), the bottom left graph reflects associations with stress (Perceived Stress Scale), and the bottom right graph reflects associations with overall mental health, assessed by taking the summation of the Patient Health Questionnaire-2, Generalized Anxiety Disorder 2-item, and Perceived Stress Scale assessments (combination score). Graphs are generated using standardized coefficients, and error bars reflect 95% CIs. The tables below the graphs in (A) and (B) display the raw coefficients (β) along with the parameters’ associated *P* value for each sleep metric association with each mental health outcome. AU: arbitrary units; combo: combination score; GAD2: Generalized Anxiety Disorder 2-item; HR: heart rate; PHQ2: Patient Health Questionnaire-2; PSS: Perceived Stress Scale; Pct: percentage.

For interpersonal associations (Figure 4A), spending a greater percent of time in zone 0 was associated with worse levels of depression, stress, and overall mental health; spending a greater percent of time in zone 1 was associated with better outcomes only for depression; and spending more time in zones 2‐4 and having higher physical activity levels were associated with better outcomes on the depression, anxiety, stress, and combined mental health assessments. For interpersonal associations (Figure 4B), an increased percent of time in zone 0 was associated with worse levels of depression, stress, and overall mental health; more percent of time in zone 1 was associated with better outcomes for depression and overall mental health; more percent of time in zone 5 was only associated with better levels of depression; and more percent of time in zones 2‐4 and being more physically active, as determined by summated HRZs and physical activity levels, both of which were associated with better levels of depression, anxiety, and stress.

#### Cross-Lagged Analysis Between Physiological Variables and Mental Health

**Figure 5.**
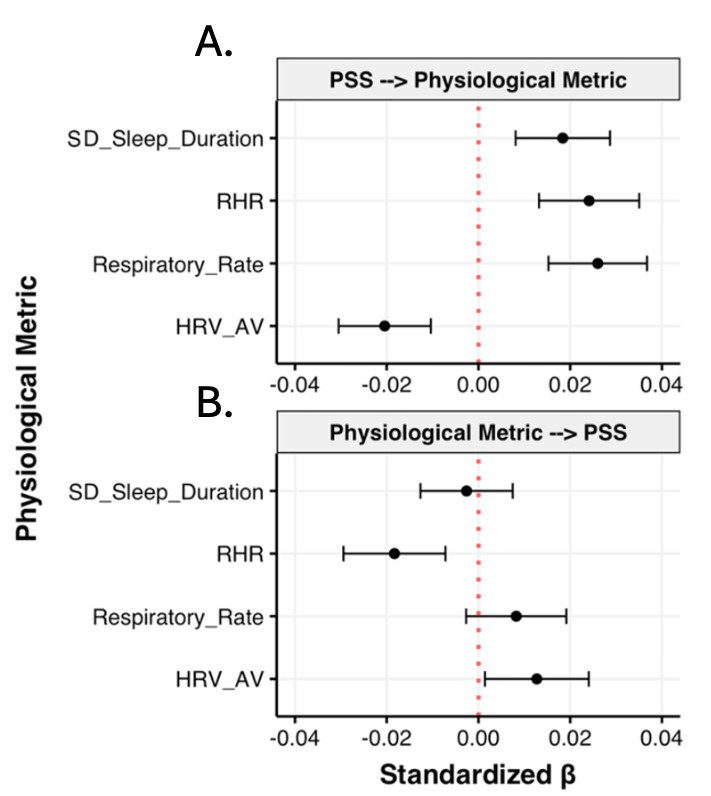
Cross-lagged analysis between Perceived Stress Scale and select cardiorespiratory and sleep metrics. (A) Effect of preceding perceived stress on following physiological metric. (B) Effect of preceding physiological metric on following perceived stress response. Error bars represent 95% CIs; “-->” in titles of graphs indicates the preceding variable in the temporal relationship. AV: average; HRV: heart rate variability; RHR: resting heart rate; PSS: Perceived Stress Scale.

To better understand the temporal relationships between mental health and physiological metrics, we leveraged structural equation models that examined both the impact of mental health state on a physiological variable and the impact of a physiological variable on mental health state. None of the lagged effects of physiological variables on mental health metrics reached significance (Multimedia Appendix 6). The effect of lagged mental state on physiological variables only reached significance when examining the effect of preceding stress on HRV_av_, respiratory rate, and RHR, whereby a higher reported stress level preceded lower HRV_av_ and higher SDs in sleep, respiratory rate, and RHR (Figure 5A). Effects of preceding physiological metrics on stress failed to reach significance (Figure 5B).

## Discussion

### Principal Findings

We conducted a comprehensive longitudinal analysis on the inter- and intrapersonal relationships between wearable-derived metrics and mental health outcomes. We find that responses to mental health surveys differ depending on age, gender, BMI, and time of year. After controlling for these demographic and seasonal variables, better mental health outcomes were found in individuals who had (1) longer sleep durations; (2) higher sleep efficiencies and HRV_av_s; (3) lower variation in their sleep patterns; (4) lower RHRs, HRV_cv_s, and respiratory rates; (5) and spent more time being physically active. Regarding intrapersonal observations, better mental health outcomes coincided with increases in sleep efficiency, sleep consistency, HRV_av_, and physical activity and decreases in RHR, respiratory rate, and HRV_cv_. Notably, changes in sleep duration displayed distinct associations with mental health outcomes when examined intra- or interpersonally, whereby an increase in sleep duration within an individual was associated with worse depression scores yet better anxiety and stress scores. Overall, this information extends our understanding of the intricate connections between physiological data and mental well-being, providing valuable insights for health care professionals, researchers, and policy makers aiming to enhance mental health interventions and personalized care strategies.

### Interpretations of Findings

Mental health exhibited seasonal variation, though the clinical meaningfulness of these fluctuations is uncertain. Some individuals report seasonal changes in mood that surpass clinically significant thresholds, such as those observed in seasonal affective disorder [26,27]. Several factors may contribute to these seasonal mood variations, including reduced sun exposure leading to changes in hormone levels (eg, melatonin and serotonin), as well as alterations in physical activity or sleep patterns [28,29]. In addition, it is noteworthy that seasonal variations have also been observed in cardiovascular metrics such as RHR, HRV, and even blood pressure [30]. Whether these seasonal changes in physiology or mood can be tempered by lifestyle modification remains uncertain but is an intriguing area of further exploration [31,32].

Individuals who slept more and had lower levels of variation in their sleep metrics displayed better scores on mental health assessments. This finding was corroborated by within-person analyses showing that increases in both sleep duration and consistency are linked with lower stress and anxiety levels. However, an interesting divergence in within- and between-person findings appears when we examine depression: while greater sleep duration was associated with lower scores of depression scores between individuals, higher depressive scores were associated with less sleep within person. One possible explanation for the differences observed in between- and within-person analyses may be due to lower amounts of sleep increasing the risk of depression [33], which would be reflected in interpersonal observations. Conversely, increases in sleep durations have been shown to be symptoms of depression [34], potentially when depression is incident with higher levels of inflammation [35], which would be reflected in intrapersonal observations. Overall, the opposing between- and within-person associations between sleep duration and depression demonstrate the importance of measuring the change in biometrics as an addition to single or sporadic measures of biometrics.

In both inter- and intrapersonal analyses, higher and more stable HRV and lower RHR values were associated with more favorable mental health profiles. The coincident changes in mental state and cardiovascular measures may stem from their interactions with the autonomic nervous system. It has been shown that individuals with higher levels of depression, anxiety, or stress tend to exhibit increased sympathetic output or diminished parasympathetic activity [36]. Regardless of the underlying mechanism, the link between RHR and mental health outcomes is well-established. For instance, a large-scale longitudinal study of over 1 million adolescent men found that a higher RHR is linked with an increased risk of psychiatric disorders [37]. Similarly, reduced HRV has been documented in psychiatric conditions such as depression, substance abuse, anxiety, and schizophrenia [38]. Although less extensively studied, day-to-day fluctuations in HRV, indexed by HRV_cv_, represent an important measure of autonomic balance with both physiological and psychological implications [39]. The association of higher HRV_av_ or lower RHR and HRV_cv_ with reduced depression, anxiety, and stress levels in our study contributes to a growing body of literature that supports the utility of tracking the cardiovascular system with wearables, as changes to these cardiorespiratory metrics may coincide with changes in mental health status.

Our results suggest that the relationship between exercise and mental health may depend upon exercise intensity. For example, the percentage of time spent in the highest intensity of exercise, zone 5, is only associated with better mental health outcomes for intrapersonal associations with depression, whereas total time spent being physically active (as determined by physical activity levels) is consistently associated with better mental health outcomes. Meta-analyses looking into the effect of interventional high-intensity exercise on mental health indicate that high-intensity exercise generally improves mental well-being [40-42]; however, too much high-intensity exercise without adequate recovery can lead to overtraining, and overtraining has been shown to be detrimental to mental health [43,44]. Meanwhile, we consistently observed that lower intensity exercise or general physical activity associates with improved mental health outcomes, which aligns with findings from previous investigations [45]. Interestingly, when comparing mental health benefits of moderate-to-vigorous continuous training (at an intensity likely to be well below HRZ 5) versus high-intensity interval training (HIIT; at an intensity likely to reach HRZ 5), moderate-to-vigorous continuous training led to significant improvements in positive affect and mental well-being over control and HIIT; furthermore, HIIT failed to improve mental health either from baseline or compared with control conditions [46]. Nevertheless, future research is warranted to better understand the potential dose-effect of high-intensity exercise on mental health.

The significant associations observed in our between- and within-person analyses but not in our cross-lagged models may be due to the temporal dynamics of physiological and psychological interactions. The mental health questionnaires (GAD2, PHQ2, and PSS) assessed symptoms over the previous 2‐4 weeks, and we aligned physiological variables with these same multiweek windows. However, psychological and physiological processes are often intertwined on much shorter timescales. Empirical evidence suggests that stress and related physiological responses (such as sleep and cardiovascular changes) are tightly linked in the short term, typically over days or even hours [47-50]. For instance, acute psychosocial stress can immediately disrupt sleep (transient “acute” insomnia), an effect that generally resolves once the stressor is removed [51]. Conversely, a single night of poor sleep can heighten stress levels and autonomic arousal (eg, elevated heart rate) the following day, whereas a good night’s sleep has the opposite effect [47]. These observations underscore the reciprocal, short-lived nature of stress-sleep interactions reported in the literature [52]. Because our analyses spanned up to 4 weeks – a time frame designed to capture recent stress exposure – any acute bidirectional effects would presumably be contained within this assessment window and potentially explain why we failed to observe more temporal relationships. Future studies using high-frequency, real-time assessments may provide further insight into the dynamic nature of these associations and help disentangle acute versus chronic effects.

### Limitations

Our findings should be interpreted in the context of this investigation’s limitations. First, selection bias may be present [53], as individuals who can afford commercial wearable devices likely represent a higher socioeconomic status. In addition, filtering for compliance within the HRZ analyses may select for a subgroup that differs from the general population. To mitigate the impact of these potential biases, we leveraged a large sample size and conducted within-person analyses. Second, our findings may be subject to recall bias since participants were asked to reflect on their mental health over the previous 2 weeks or month at the time of completing surveys. To address this, future studies could incorporate ecological momentary assessments, allowing for real-time data collection to minimize recall inaccuracies. Third, events or environmental factors not measured in our study may influence the relationship between mental health and wearable data. To partly mitigate this limitation, we leveraged data from a large and diverse sample spanning multiple countries, potentially reducing the impact of localized events or environmental factors. Fourth, we did not control for menstrual phase when analyzing cardiovascular or survey data in female participants, which may introduce variability. Future studies could incorporate menstrual cycle tracking to control for physiological variations related to menstrual phases. Fifth, this investigation specifically measured stress, anxiety, and depression using the PSS, GAD2, and PHQ2 scales and did not examine other mental health disorders such as bipolar disorder or post-traumatic stress disorder. We refrained from including additional diagnostic surveys to avoid increasing participant burden and reducing study retention. However, future research may benefit from incorporating broader diagnostic measures or clinical evaluations to more comprehensively capture mental health outcomes.

### Conclusion

Leveraging a large sample size, longitudinal design, and continuous second-by-second data, this study provides valuable insights into the relationships between wearable-derived physiological metrics and mental health outcomes. The associations observed across multiple biometric indicators suggest that wearables hold promise as complementary tools in mental health monitoring. As our understanding of the dynamic interplay between physiological and psychological states continues to evolve, this research adds to the growing body of evidence that wearables can help elucidate the intricate relationship between mental states and physiological processes. Future research should further explore how wearables can be leveraged in real-time assessments and adaptive interventions to enhance early detection, prevention, and treatment of mental health conditions.

## Supplementary material

10.2196/64955 Multimedia Appendix 1 Mental Health Questionnaires.

10.2196/64955 Multimedia Appendix 2 Metric calculations.

10.2196/64955 Multimedia Appendix 3 Simplified Cross-lagged model. Boxes = measurements; black dashed paths = autoregressive effects; black curved paths = covariance; red solid path = cross-lagged effects. tn represents an initial timepoint and tn+1 reflects the subsequent timepoint. Error terms and covariates omitted for visualization purposes.

10.2196/64955 Multimedia Appendix 4 Associations between mental health and age, gender, BMI, and season. Associations between (a) age and gender, (b) BMI, and (c) season. For (a), (b), and (c), the top left graph reflects the associations with depression (PHQ2), the top right graph reflects associations with anxiety (GAD2), the bottom left graph reflects associations with stress (PSS), and the bottom right graph reflects associations with overall mental health, assessed by taking the summation of the PHQ2, GAD2, and PSS assessments (Combo). The bars in (b) represent the average mental health score when binning BMI into underweight (<18.5 kg/m2), healthy weight (≥18.5 kg/m2 and <25 kg/m2), overweight (≥25 kg/m2 and <30 kg/m2), and obese (≤30 kg/m2). The bars in (c) represent the average mental health score when binning time of year into seasons (i.e., winter, spring, summer, and fall). Error bars reflect 95% confidence intervals. Abbreviations: PHQ2 = Patient Health Questionnaire-2; GAD2 = Generalized Anxiety Disorder 2-item; PSS = Perceived Stress Scale; BMI = Body Mass Index.

10.2196/64955 Multimedia Appendix 5 Pairwise comparisons for BMI and Season. (a) The pairwise comparisons between BMI classifications. (b) The pairwise comparisons between seasons.

10.2196/64955 Multimedia Appendix 6 Cross-lagged analysis between mental health outcomes and physiological metrics. Effect of preceding mental health outcome on following physiological metric. Error bars represent 95% confidence intervals; “-->” in titles of graphs indicated the preceding variable in the temporal relationship. .

## Abbreviations

GAD2: Generalized Anxiety Disorder 2-item
HIIT: high intensity interval training
HRV: heart rate variability
HRV_av_: average heart rate variability
HRV_cv_: coefficient of variation of heart rate variability
HRZ: heart rate zone
PHQ2: Patient Health Questionnaire-2
PSS: Perceived Stress Scale
RHR: resting heart rate
