# Supplementary material for: Inter- and Intrapersonal Associations Between Physiology and Mental Health: A Longitudinal Study Using Wearables and Mental Health Surveys
Source: J Med Internet Res. 2025 Jul 23;27:e64955. doi: 10.2196/64955 (PMC12310073; doi:10.2196/64955)
Supplement: Multimedia Appendix 1 [file jmir-v27-e64955-s001.docx]

The following document outlines how data were collected for mental health research via WHOOP Inc’s digital platform:

**WHOOP mental health research**

Complete the following questions once per month to help advance mental health research.

**[Perceived Stress Scale]**

Answer choices and their numerical mapping:

- Never = 0
- Almost Never = 1
- Sometimes = 2
- Fairly Often = 3
- Very Often = 4

Reverse scores for questions d, e, g, h:

- Never = 4
- Almost Never = 3
- Sometimes = 2
- Fairly Often = 1
- Very Often = 0

1. In the past month:
   1. How often have you been upset because of something that happened unexpectedly?
      1. Never
      2. Almost Never
      3. Sometimes
      4. Fairly Often
      5. Very Often
   2. How often have you felt that you were unable to control the important things in your life?
      1. Never
      2. Almost Never
      3. Sometimes
      4. Fairly Often
      5. Very Often
   3. How often have you felt nervous and stressed?
      1. Never
      2. Almost Never
      3. Sometimes
      4. Fairly Often
      5. Very Often
   4. How often have you felt confident about your ability to handle your personal problems?
      1. Never
      2. Almost Never
      3. Sometimes
      4. Fairly Often
      5. Very Often
   5. How often have you felt that things were going your way?
      1. Never
      2. Almost Never
      3. Sometimes
      4. Fairly Often
      5. Very Often
   6. How often have you found that you could not cope with all the things that you had to do?
      1. Never
      2. Almost Never
      3. Sometimes
      4. Fairly Often
      5. Very Often
   7. How often have you been able to control irritations in your life?
      1. Never
      2. Almost Never
      3. Sometimes
      4. Fairly Often
      5. Very Often
   8. How often have you felt that you were on top of things?
      1. Never
      2. Almost Never
      3. Sometimes
      4. Fairly Often
      5. Very Often
   9. How often have you been angered because of things that were outside of your control?
      1. Never
      2. Almost Never
      3. Sometimes
      4. Fairly Often
      5. Very Often
   10. How often have you felt difficulties were piling up so high that you could not overcome them?
       1. Never
       2. Almost Never
       3. Sometimes
       4. Fairly Often
       5. Very Often

**[Patient Health Questionnaire 2]**

Answer choices and their numerical mapping

- Not at all = 0
- Several days = 1
- More than half of the days = 2
- Nearly every day = 3

1. In the past two weeks, have you:
   1. Had little interest or pleasure in doing things?
      1. Not at all
      2. Several days
      3. More than half of the days
      4. Nearly every day
   2. Felt down, depressed or hopeless?
      1. Not at all
      2. Several days
      3. More than half of the days
      4. Nearly every day

**[Generalized Anxiety Disorder 2-Item]**

Answer choices and their numerical mapping

- Not at all = 0
- Several days = 1
- More than half of the days = 2
- Nearly every day = 3

1. In the past two weeks, have you:
   1. Felt nervous, anxious or on edge?
      1. Not at all
      2. Several days
      3. More than half of the days
      4. Nearly every day
   2. Not been able to stop or control worrying?
      1. Not at all
      2. Several days
      3. More than half of the days
      4. Nearly every day
