# Supplementary figures and images for: Inter- and Intrapersonal Associations Between Physiology and Mental Health: A Longitudinal Study Using Wearables and Mental Health Surveys
Source: J Med Internet Res. 2025 Jul 23;27:e64955. doi: 10.2196/64955 (PMC12310073; doi:10.2196/64955)

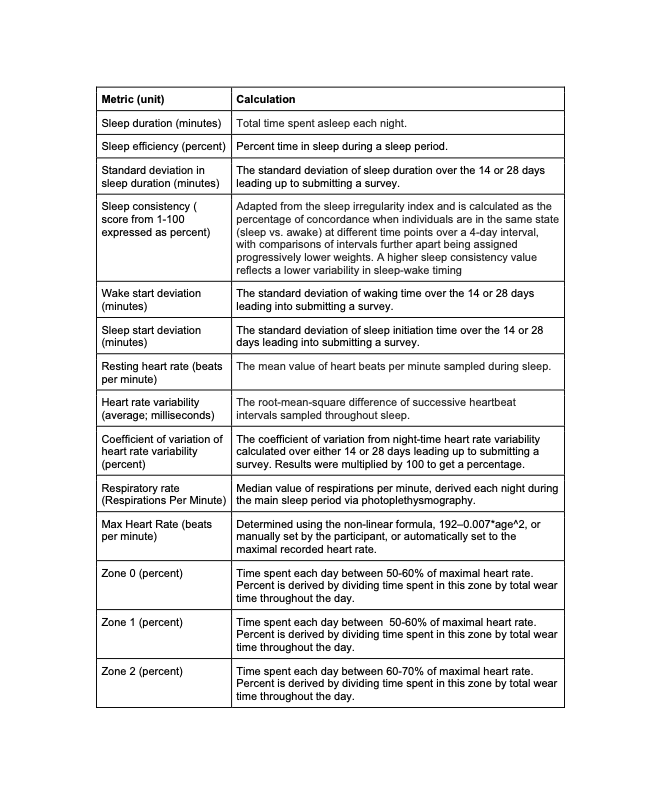

Supplement: Multimedia Appendix 2 [file jmir-v27-e64955-s002.png]

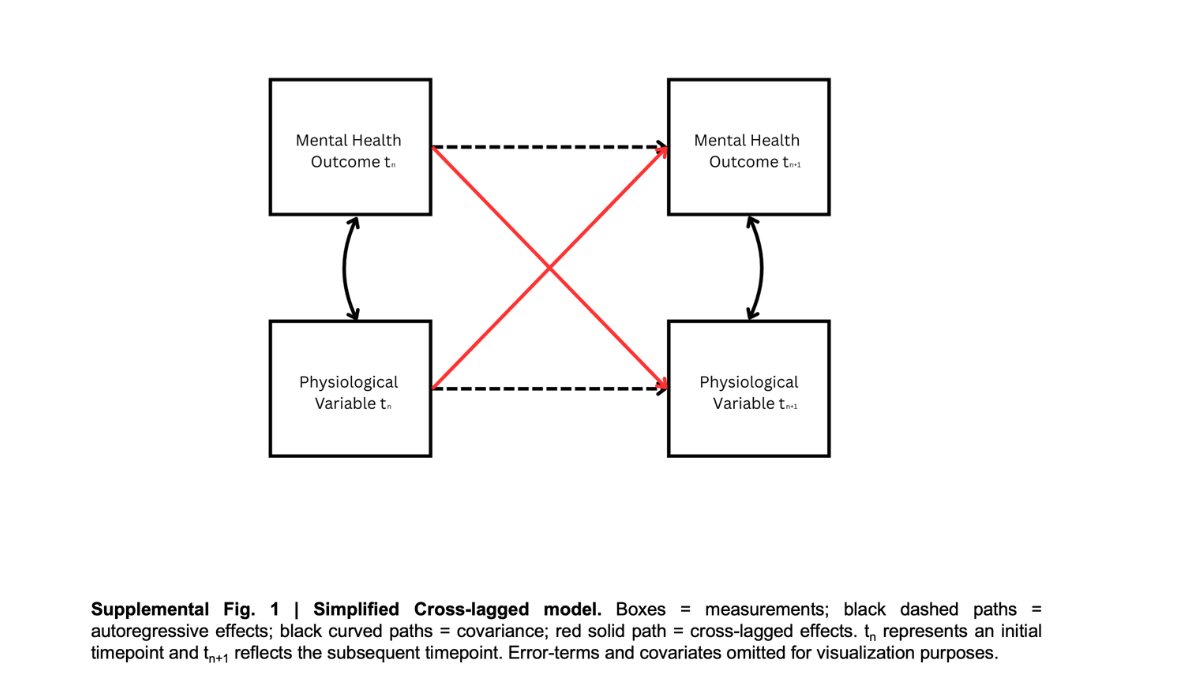

Supplement: Multimedia Appendix 3 [file jmir-v27-e64955-s003.png]

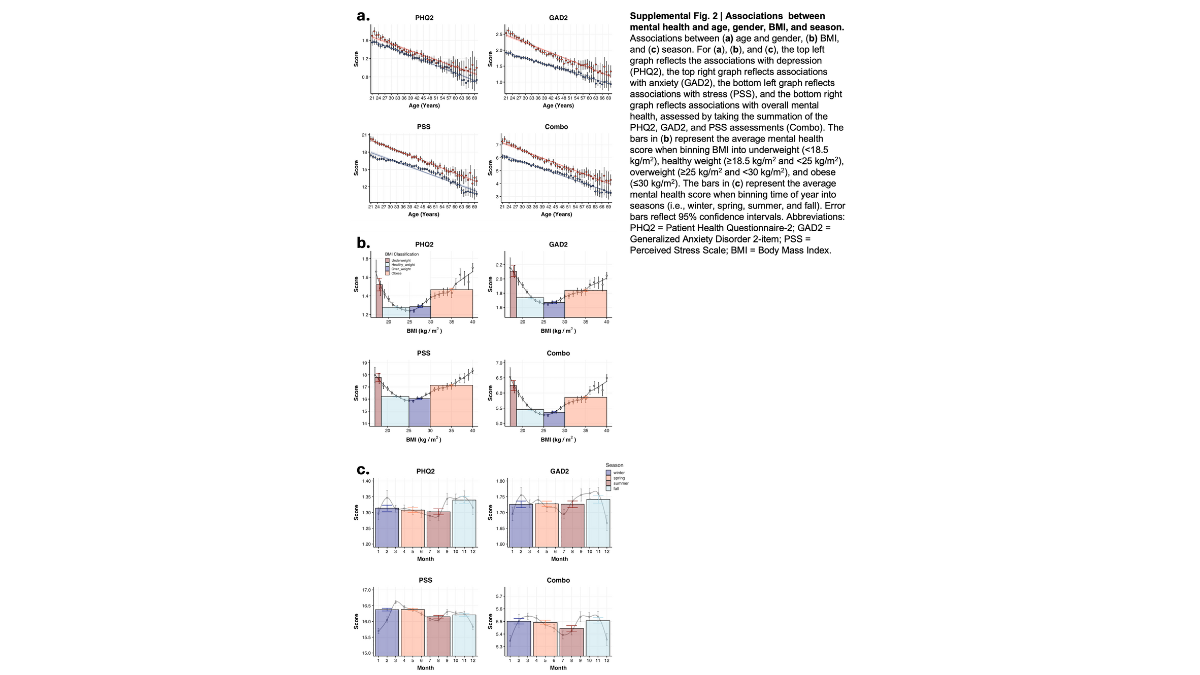

Supplement: Multimedia Appendix 4 [file jmir-v27-e64955-s004.png]

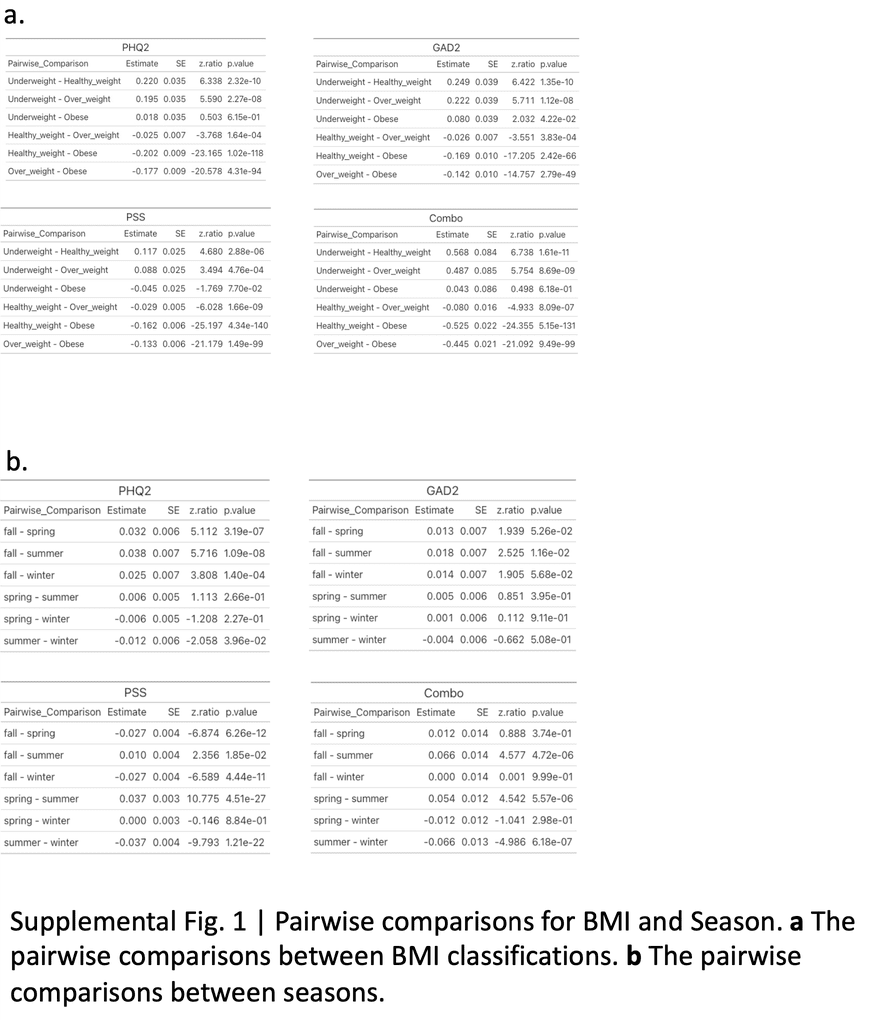

Supplement: Multimedia Appendix 5 [file jmir-v27-e64955-s005.png]

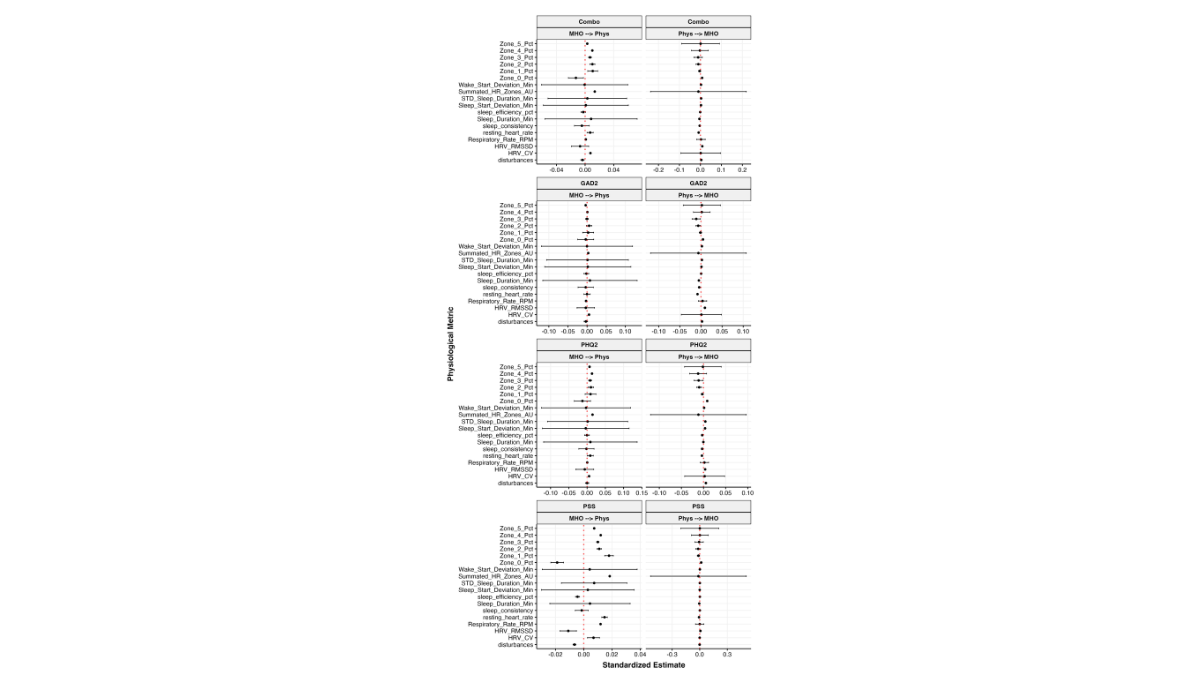

Supplement: Multimedia Appendix 6 [file jmir-v27-e64955-s006.png]
